# Equine Herpesvirus Infections: Treatment Progress and Challenges in Horses and Donkeys

**DOI:** 10.3390/vetsci12111082

**Published:** 2025-11-13

**Authors:** Muhammad Zahoor Khan, Yanfei Ji, Xuewei Fan, Yihong Liu, Wenqiang Liu, Changfa Wang

**Affiliations:** 1 College of Agriculture and Biology, Liaocheng University, Liaocheng 252000, China; zahoorkhan@lcu.edu.cn (M.Z.K.);; 2 College of Veterinary Medicine, Northeast Agricultural University, Harbin 150030, China; 3 Heilongjiang Agricultural Economy Vocational College, Mudanjiang 157041, China

**Keywords:** equine herpesvirus, donkey, horses, pathogenesis, glycoproteins, vaccination, global epidemiology

## Abstract

Equine herpesvirus (EHV) infections represent a significant global veterinary and economic challenge affecting both horses and donkeys across all inhabited continents. This narrative review comprehensively examines the nine distinct EHV species (EHV-1 through EHV-9), their taxonomic classification within Alphaherpesvirinae and Gammaherpesvirinae subfamilies, and their diverse host tropism patterns. The complex molecular pathogenesis involves sophisticated viral glycoproteins (gK, gB, gC, gH, gM, gL, gG, gD, gI, gE) that orchestrate cellular invasion, immune evasion, and intercellular transmission. Clinical manifestations vary considerably, ranging from respiratory diseases and reproductive failures to severe neurological disorders, with EHV-1 demonstrating the most severe presentations including myeloencephalopathy. Global distribution analysis reveals widespread circulation across Europe, Asia, Africa, the Americas, and Oceania, with species-specific clinical patterns. Current therapeutic options remain largely supportive, with experimental compounds like berbamine and cepharanthine, celastrol, blebbistatin, and hyperoside showing promise in preclinical studies. Vaccination programs demonstrate limited effectiveness, failing to prevent transmission at population levels despite inducing individual immune responses. The sophisticated immune evasion strategies employed by EHVs, including the “Trojan horse” mechanism utilizing infected leukocytes, highlight the complexity of host–pathogen interactions and underscore the urgent need for innovative prevention and treatment strategies.

## Simple Summary

Equine herpesviruses pose a persistent global threat to horse and donkey populations, with nine identified species causing diverse health complications. These pathogens exhibit notable adaptability via specialized glycoprotein networks that mediate cellular and immune evasion. Clinical presentations span from mild respiratory symptoms to fatal neurological conditions, with EHV-1 representing the most dangerous variant. Despite worldwide surveillance efforts documenting cases across five continents, effective interventions remain elusive. Current vaccines provide individual protection but fail to control population-level transmission. Emerging therapeutic research focuses on natural compounds targeting viral replication stages, yet comprehensive disease management requires integrated approaches combining improved diagnostics, enhanced biosecurity measures, and novel treatment strategies.

## 1. Introduction

Equids, comprising horses (*Equus caballus*) and donkeys (*Equus asinus*), represent economically and culturally significant livestock species with diverse applications across global agricultural systems [1,2]. Donkeys serve as essential working animals in developing regions while also contributing to specialized markets through donkey milk production, which is valued for its nutritional properties and therapeutic applications in human medicine [3,4,5,6,7,8]. Additionally, donkey meat consumption occurs in certain regions [9,10,11,12], and the production of ejiao—a traditional Chinese medicine derived from donkey hide gelatin—has created substantial economic demand [13,14,15]. Horses maintain their historical importance in agriculture as working animals while simultaneously supporting multi-billion-dollar industries including racing, breeding, and recreational activities [1,16,17]. This intimate and extensive human–equid relationship, characterized by close contact during husbandry, training, and recreational activities, necessitates comprehensive understanding of equine diseases that may impact both animal welfare and human health [18,19,20].

**Figure 1:**
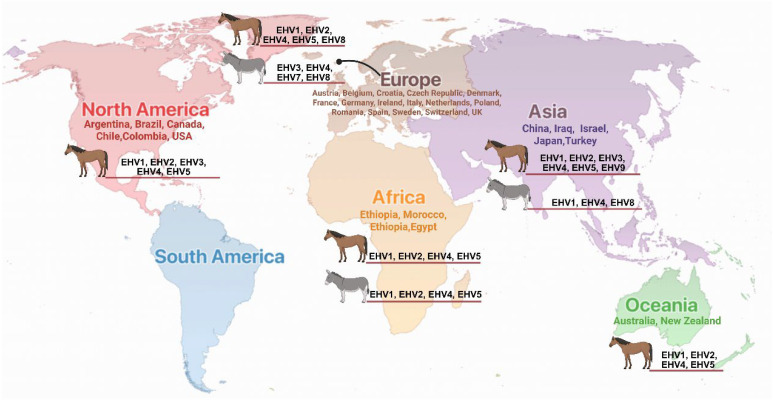
Global distribution of equine herpes virus infections in horses and donkeys. Countries with documented EHV infections are listed by continent with corresponding viral types (EHV-1 through EHV-9) identified in each region. Continental regions are color-coded for clarity.

Among the infectious diseases affecting equids, equine herpesvirus (EHV) infections represent a significant veterinary and economic concern due to their widespread distribution, diverse clinical manifestations, and potential for causing substantial morbidity and mortality in affected populations (Figure 1) [21,22,23,24,25,26,27]. EHV infection poses a significant therapeutic challenge due to the consistently minimal response to both treatment and vaccination in equines [22,28]. The Equidae family is susceptible to infection by several distinct herpesvirus species, with equine herpesvirus-1 (EHV-1), EHV-2, EHV-3, EHV-4, EHV-5 and EHV-8 being the most clinically relevant pathogens [29,30,31,32,33,34,35,36]. EHV infections are economically important because they cause respiratory disease, abortion storms, reproductive failure, neonatal mortality, and neurological disease known as equine herpesvirus myeloencephalopathy (EHM) [21,37]. The clinical manifestations of EHV infections vary considerably depending on the viral strain, host factors including age and immune status, and environmental stressors [38,39,40,41,42]. The economic impact of EHV infections extends beyond direct treatment costs to include losses from reduced productivity, quarantine measures, event cancellations, and trade restrictions, particularly affecting the racing and breeding industries. Current diagnostic approaches have evolved significantly with the advent of molecular techniques, allowing for rapid and accurate identification of viral nucleic acids and strain differentiation. However, challenges remain in distinguishing between active infection and latent carriage, particularly in the context of disease outbreaks and movement regulations [43,44]. Treatment options remain largely supportive, emphasizing the critical importance of prevention through vaccination programs and management practices [45].

This review provides a comprehensive overview of equine herpesvirus infections in horses and donkeys, examining the viral etiology, clinical presentations, diagnostic approaches, treatment strategies, and prevention measures. Understanding the complexities of EHV infections is crucial for developing effective control programs that protect both equine populations and the economic interests dependent upon these valuable animals.

## 2. The Classification of EHVs and Their Associated Glycoproteins

The EHVs constitute a taxonomically diverse group comprising nine distinct viral species, each characterized by specific phylogenetic relationships and host tropism patterns (Table 1). Within the Alphaherpesvirinae subfamily, several EHV species are classified under the genus Varicellovirus, including EHV-1, EHV-3, EHV-4, EHV-6, EHV-8, and EHV-9 [46]. In contrast, EHV-2, EHV-5 and EHV-7 belong to the Gammaherpesvirinae subfamily, genus Percavirus [47,48]. Host specificity varies among these viral species, with EHV-1, EHV-4, EHV-5, and EHV-8 demonstrating broad equid tropism affecting both horses and donkeys, while EHV-2 similarly infects horses and donkeys, and EHV-7 exhibits preferential infection of donkeys with secondary horse susceptibility, and EHV-3 and EHV-9 display restricted host specificity limited to horses.

The molecular pathogenesis of equine herpesvirus (EHV) infections involves a sophisticated and complex array of viral glycoproteins that collectively orchestrate the infection process (Table 2) [68]. Each glycoprotein is encoded by specific open reading frames (ORFs) within the viral genome and serves distinct yet interconnected functional roles in mediating viral–host interactions [46]. These proteins work synergistically to establish successful infection, facilitate viral replication, and ensure efficient transmission between host cells. The comprehensive glycoprotein repertoire encompasses several key components, each possessing highly specialized functions that contribute to the overall pathogenic strategy.

Glycoprotein gK (ORF6) serves as a pivotal orchestrator of host cell invasion, functioning as the primary mediator that initiates the infection cascade [69]. Furthermore, this protein facilitates critical viral replication processes within the host cell nucleus and subsequently mediates intercellular viral transmission through complex molecular interactions with cellular receptors. In addition to gK, glycoprotein gB (ORF33) functions as a multifunctional protein that is absolutely essential for successful cellular invasion [70,71]. This protein not only enables efficient viral dissemination between neighboring host cells but also facilitates antigenic epitope presentation, thereby playing a dual role in both infection propagation and immune system evasion strategies. Complementing these invasion mechanisms, glycoprotein gC (ORF16) contributes significantly to host cell invasion processes through its specialized binding domains [72,73]. Moreover, this protein enhances epitope recognition capabilities and serves as a crucial determinant of viral virulence factors. The synergistic action of gC with other glycoproteins amplifies the overall infectivity of the viral particle. Similarly, glycoprotein gH (ORF39) is specifically required for efficient host cell invasion and works in concert with other envelope proteins to facilitate membrane fusion processes [74]. Concurrently, glycoprotein gM (ORF52) assumes a critical role in facilitating viral transmission between neighboring host cells, particularly during the later stages of infection when cell-to-cell spread becomes predominant [74]. The glycoprotein network extends further to include gL (ORF62), which actively participates in cellular invasion processes through its interaction with cellular membrane components [75]. Additionally, glycoprotein gG (ORF70) assumes responsibility for epitope presentation and sophisticated immune system modulation, enabling the virus to evade host immune surveillance mechanisms effectively [76].

Furthermore, glycoprotein gD (ORF72) demonstrates remarkable versatility by mediating both host cell invasion and epitope presentation functions [77]. This dual functionality makes gD a particularly important target for therapeutic interventions. The EHV-1 and EHV-4 gDs interact with equine major histocompatibility complex I (MHC-I) to initiate entry into equine cells [78,79]. Finally, the coordinated action of glycoproteins gI (ORF73) and gE (ORF74) collectively facilitates intercellular viral transmission through specialized cell-to-cell spread mechanisms that bypass extracellular immune recognition.

These glycoproteins, operating as an integrated molecular network, collectively constitute the fundamental framework underlying viral pathogenesis. Consequently, they are essential mediators of host–pathogen interactions and serve as primary modulators of immune response mechanisms. The intricate interplay between these proteins determines the overall success of viral infection and influences disease outcomes in affected hosts.

**Table 2:** Major EHV glycoproteins and their functions.

Gene Sequence	Encoded Protein	Main Functions	References
ORF6	gK	Invasion of host cells, viral replication, viral transmission between host cells	[69]
ORF33	gB	Invasion of host cells, transmission of viruses between host cells, epitopes	[70,71,72,73,80,81]
ORF16	gC	Invasion of host cells, epitopes, viral virulence	[71,72,73]
ORF39	gH	Invasion of host cells	[60,74]
ORF52	gM	Virus transmission between host cells	[74,82,83]
ORF62	gL	Invasion of host cells	[75]
ORF70	gG	Epitopes Immune modulation	[76,84,85,86]
ORF72	gD	Invasion of host cells, epitopes	[52,71,87]
ORF73	gI	Virus transmission between host cells	[88,89,90,91]
ORF74	gE	Virus transmission between host cells	

## 3. Global Distribution and Clinical Manifestations of Herpes Virus Infections in Donkeys and Horses

Herpes virus infections represent a significant health concern in equids worldwide, affecting both horses and donkeys across all inhabited continents. The documented cases span from Europe to Oceania, demonstrating the global distribution and diverse clinical presentations of these viral pathogens (Table 3). This comprehensive analysis presents the geographic distribution patterns and clinical manifestations of various EHV types based on reported cases from multiple countries.

Europe demonstrates the highest diversity of reported herpes virus infections, with cases documented across 15 countries. The most frequently reported virus types include EHV-1, EHV-2, EHV-4, and EHV-5, with notable clinical presentations ranging from respiratory diseases to neurological manifestations. Countries such as Germany, Italy, and the Netherlands have reported multiple virus types, indicating either higher surveillance capacity or increased viral circulation [57,92,93]. The Asian continent demonstrates significant herpes virus activity, particularly in China, where multiple virus types (EHV-1, EHV-4, and EHV-8) have been identified in both horses and donkeys. Notably, EHV-8 infections in donkeys have been associated with severe clinical outcomes including respiratory disease, abortion, and neurological disorders [61,62,94]. Japan shows a unique pattern with reports of EHV-3, EHV-4, and EHV-9 in horses [63,95]. While in Korea, cases of EHV2 and EHV5 in horses have been reported [96].

African countries, particularly Ethiopia and Morocco, show a concerning pattern of herpes virus infections affecting both horses and donkeys. Ethiopia reports a diverse range of virus types (EHV-1, EHV-2, EHV-4, EHV-5) with clinical manifestations including abortion, respiratory distress, and neurological signs [34,97,98,99]. The North and South American continents demonstrate varied patterns of infection, with reports extending from Canada to Chile, while Oceania contributes additional cases from Australia and New Zealand, completing the global distribution pattern.

Among the various virus types, EHV-1 demonstrates the most severe clinical presentations globally, consistently associated with myeloencephalopathy, viremia, and nasal discharge across multiple continents [100]. Neurological manifestations are particularly prominent in reports from France, Italy, Spain, Chile, and the USA [49,101,102,103,104,105,106]. In contrast, EHV-4 primarily causes respiratory tract infections, with documented cases showing pyrexia, nasal discharge, mandibular lymphadenopathy, and increased lung sounds. This pattern remains consistent across Europe, Asia, and Africa [59,107]. EHV-2 and EHV-5 infections show particular association with respiratory diseases and, notably, squamous cell carcinoma in horses. EHV-2/5 DNA has been detected in horses with head-neck, ocular, penile, and vulvar squamous cell carcinoma, suggesting potential oncogenic properties [108,109]. In addition, while EHV-2/5 DNA has been detected in equine squamous cell carcinoma cases, causality remains unproven. Future studies should employ experimental inoculation models in immunocompromised horses with careful control for confounding factors including UV exposure, genetic predisposition, and co-infections with equine papillomaviruses to definitively establish the oncogenic potential of these gammaherpesviruses. EHV-8 shows a distinctive pattern of causing abortion in pregnant mares and has been associated with respiratory disease and neurological disorders in donkeys, particularly in Asian populations [31,57].

The data reveals distinct susceptibility patterns between horses and donkeys. While horses are susceptible to the full spectrum of EHV types (EHV-1 through EHV-9), donkeys show particular susceptibility to EHV-1, EHV-4, EHV5, EHV7 and EHV-8, AHV types. Regarding asinine herpesviruses (AHV), AHV-1, AHV-5, and AHV-7 appear to be donkey-specific with AHV-1&5 causing severe interstitial pleuropneumonia and pulmonary fibrosis [47,92]. The majority of diagnoses were confirmed through PCR-based methods, demonstrating the reliability of molecular diagnostics for herpes virus identification. Some studies employed ELISA-based serological testing, particularly in African countries, while postmortem pathological findings provided crucial diagnostic information in severe cases [92,110]. This global overview demonstrates the widespread distribution and significant clinical impact of herpes virus infections in equids. The diversity of clinical presentations, from mild respiratory signs to severe neurological manifestations and reproductive failures, underscores the importance of continued surveillance and research into these viral pathogens. The geographic distribution patterns suggest both endemic circulation and possible cross-border transmission, highlighting the need for coordinated international monitoring and control strategies. The documented evidence across five continents confirms that herpes virus infections in equids constitute a global veterinary health challenge requiring sustained scientific attention and collaborative international response efforts.

**Table 3:** Global distribution of equine herpes virus and their clinical signs in donkeys and horses.

Europe				
Virus Type	Species	Reported Clinical Signs	Country	Reference
EHV2/5	Horses	Herpesvirus DNA (EHV2 and EHV5) was detected in horses with head-neck, ocular, penile, and vulvar squamous cell carcinoma respectively.	Austria	[108,111]
EHV1	Horses	Respiratory disorders, abortion and neonatal foal death	Belgium	[112]
EHV1/4	Horses	Abortion	Bulgaria	[113]
EHV-1	Horses	Abortion and neonatal foal death	Croatia	[114]
EHV-2	Horses	Keratitis and keratoconjunctivitis	Czech Republic	[115]
EHV4	Horses	Pyrexia, nasal discharge, mandibular lymphadenopathy and increased lung sound upon auscultation	Denmark	[59]
EHV1	Horses	Myeloencephalopathy and fever	France	[102,116]
EHV4	Horses	Respiratory diseases (PCR diagnosis)	Germany	[117]
EHV1	Horses	Immune suppression, abortion and respiratory diseases	Germany	[118,119]
EHV1/4	Horses	Rhinopneumonitis and abortion	Germany	[120]
EHV2	Horses	Keratoconjunctivitis	Germany	[121]
EHV-8	Horses	Abortion in pregnant mares	Ireland	[31]
AHV-1&5	Donkeys	Interstitial pleuropneumonia and pulmonary fibrosis (Postmortem findings)	Italy	[92]
AHV7&5	Donkeys	Respiratory distress (increased respiratory rate, nostril flaring, nasal discharge and pyrexia)	Italy	[47]
EHV3	donkeys	Ulcerative stomatitis	Italy	[30]
EHV-1	Horses	Myeloencephalopathy, Viremia, with nasal discharge (PCR diagnosis)	Italy	[49]
EHV1,4/5	Horses	Abortion and neurological disorders.	Italy	[122]
EHV-8	Donkeys	Respiratory disease, abortion and neurological disorders.	Netherlands	[57]
EHV1/4	Horses	Acute respiratory disease, abortion and neurological signs (using PCR)	Netherlands	[123,124,125]
EHV1	Horses	Abortion, neonatal foal death	Poland	[126,127]
EHV-4	Donkeys	Upper respiratory tract infection, abortion and neurological signs	Romania	[107]
EHV-1	Horses	Myeloencephalopathy and Lymphopenia	Spain	[128,129]
EHV2	Horses	Immunosuppression and upper respiratory tract infection	Sweden	[130]
EHV5	Horses	Equine Multinodular Pulmonary Fibrosis with Leukocytosis, hyperfibrinogenemia and hypoxemia. Thoracic radiographs showed pneumonia with a multifocal nodular pattern	Sweden	[131,132]
EHV-1	Horses	Abortion and myeloencephalopathy	Switzerland	[133]
EHV-1/4	Horses	Rhinopneumonitis, abortions, paresis and neonatal foal deaths.	UK	[134,135]
Asia				
EHV-1	Donkeys	Respiratory distress, abortion and death of young foal	China	[50]
EHV-1	Donkeys	Abortion and neurological signs	China	[136]
EHV-1	Horses	Respiratory distress and abortion	China	[137,138]
EHV-1&4	Donkeys	Abortion and respiratory signs	China	[58]
EHV-8	Donkeys	Respiratory disease, abortion and neurological disorders.	China	[139,140,141]
			Israel	
EHV2,4,5	Horses	Respiratory disease (increased respiratory rate, nasal dis-charge and pyrexia)	China	[142]
EHV1	Donkeys and horses	Respiratory disease (increased respiratory rate, nasal dis-charge and pyrexia)	Iraq	[143]
EHV1/4	Horses	Respiratory diseases and fever	Israel	[144]
EHV 3	Horses	Equine coital exanthema followed formation of papules, pustules, ulcers and scabs on the progenital skin	Japan	[95]
EHV 4	Horses	Nasal discharge, mucosal inflammation of upper respiratory tract and enlargement of mandibular lymph node.	Japan	[145]
EHV 9	Horses	Fever and respiratory distress	Japan	[63]
EHV-1	Horses	Neurological disorders	Japan	[146]
EHV4	Horses	equine rhinopneumonitis	Japan	[147]
EHV-1&4	Donkey and horses	Abortion and respiratory signs	Turkey	[148,149]
EHV5	Horses	Respiratory disease (increased respiratory rate, nasal discharge and pyrexia)	Turkey	[48]
Africa				
EHV-1	Donkeys	Abortion and neurological signs	Ethiopia	[150]
EHV-1&4	Donkeys and horses	Abortion and respiratory signs	Morocco, Ethiopia	[98,151,152]
EHV1,2&5	Donkeys and horses	Respiratory distress (increased respiratory rate, nostril flaring, nasal discharge and pyrexia)	Ethiopia	[99]
EHV-2/5	Donkeys and horses	Respiratory distress (increased respiratory rate, nostril flaring, nasal discharge and pyrexia)	Ethiopia	[65]
EHV-5	Donkeys	Abortion, respiratory distress and neurological signs	Ethiopia	[47,97]
EHV1/4	Horses	Respiratory diseases and neurological signs (ELISA diagnose)	Morocco	[110]
EHV4	Horses	Abortion	Egypt	[153]
North and South America				
EHV1	Horses	Abortions, perinatal foal mortality, and myeloencephalopathy	Argentina	[154,155]
EHV2	Horses	Immunosuppression in foals, upper respiratory tract disease, conjunctivitis, general weakness	Argentina	[156]
EHV2	Horse	Respiratory distress (increased respiratory rate, nostril flaring, nasal discharge and pyrexia)	Brazil	[64]
EHV1/4	Horses	Abortion and respiratory distress	Brazil	[157,158]
EHV1	Horses	Myeloencephalopathy and nasal discharge	Canada	[159]
EHV-1	Horses	Myeloencephalopathy, Viremia, with nasal discharge (PCR diagnosis)	Chile	[101]
EHV-3	Horses	Equine coital rash (ECE) (PCR diagnosis)	Chile	[53]
EHV1/4	Horses	Abortion and respiratory distress (Indirect ELISA and PCR)	Colombia	[160]
EHV3	Horses	Equine coital exanthema	Columbia	[161]
EHV-5	Horses	Facial lymphohistiocytic interface dermatitis	USA	[109]
EHV5	Horses	Equine multinodular pulmonary fibrosis and lymphoma	USA	[162,163]
EHV1	Horses	Myeloencephalopathy, Viremia, with nasal discharge	USA	[164,165,166]
Oceania				
EHV1	Horses	Idiopathic hemorrhagic cystitis	Australia	[167,168]
EHV1,2,4/5	Horses	Respiratory distress (increased respiratory rate, nostril flaring, nasal discharge and pyrexia)	Australia	[169,170,171]
EHV2, EHV5	Horses	Respiratory disease (increased respiratory rate, nasal discharge and pyrexia)	Australia, New Zealand	[172,173,174]

## 4. The Pathogenesis of and Immune Response to EHVs

The pathogenesis of equine herpesviruses represents a complex interplay between viral invasion strategies and host immune responses that ultimately determines disease outcome. EHV initiates infection in the upper respiratory tract by exploiting innate immune mechanisms, particularly co-opting antimicrobial equine β-defensins (eBDs) to enhance binding and infection of primary respiratory epithelial cells (ERECs). This process is facilitated by viral glycoprotein gM, which stabilizes the virion envelope and confers resistance to eBD-mediated permeabilization [175]. Despite this enhanced entry mechanism, EHV replication in the respiratory epithelium remains inherently limited, representing a strategic viral mechanism to control local innate immune responses while facilitating establishment of latency in trigeminal ganglion neurons and modulating leukocyte recruitment [176,177,178,179].

Following respiratory tract infection, viral shedding continues for approximately seven days, accompanied by virus migration to local lymph nodes, establishment of leukocyte-associated viremia, and subsequent dissemination to multiple organ systems [180,181,182]. Studies utilizing EHV-8 in murine models have revealed multi-organ tropism with viral DNA detection in lungs, liver, brain, and intestinal tissues [62]. The pulmonary system appears particularly susceptible, with EHV-8 demonstrating preferential replication in lung tissue and triggering significant elevation of pro-inflammatory cytokines including IL-1β, IL-6, IL-8, and IFN-α, indicating the virus’s capacity to induce localized inflammatory responses [62,183].

A validated “old mare model” using horses > 18 years old to experimentally induce equine herpesvirus myeloencephalopathy (EHM) demonstrated that all 9 old mares developed neurological disease compared to only 1 of 9 young horses [184]. Protection from EHM was associated with early, robust type I interferon responses and TH-1 cellular immunity (including increased IFN-α, IL-1β, IFN-γ, and CXCL10), while horses that developed EHM showed delayed interferon responses, higher regulatory cytokines (IL-10, TGF-β), and elevated IgG3/5 antibodies indicative of a TH-2-biased immune response. The EHM horses also exhibited significantly higher cell-associated viremia but reduced nasal viral shedding compared to protected animals, suggesting that the failure to mount timely innate immune responses at the respiratory tract enables systemic viral dissemination and neurological disease [184].

### 4.1. Innate Immune Responses and Viral Modulation

The host immune response to EHV infection involves activation of multiple signaling pathways and inflammatory mediators. Transcriptomic analysis has revealed that EHV-8 modulates host immune responses through TNF pathway activation, involving key targets such as TNFR1, NF-κB p65, and MAP3K8, along with inflammatory signaling pathways including TNF and NF-κB signaling [61]. The type I interferon system serves as a critical antiviral defense mechanism, with EHV-1 infection upregulating IFN-α in ERECs and mucosal explants [185]. However, viral modulation of this response occurs in a strain-specific manner, with neurovirulent strains demonstrating sensitivity to IFN restriction while non-neurovirulent strains have evolved effective anti-IFN mechanisms to prioritize upper respiratory tract replication [185].

EHV-1 infection triggers a broader innate immune response characterized by upregulation of Toll-like receptors (TLR-3, TLR-9), inflammatory cytokines (IL-1, TNF-α, IL-6), and key chemokines including IL-8, MCP-1/CCL2, and CCL5 to recruit target immune cells such as CD172a+ monocytes and T lymphocytes to infection sites [186,187]. Neurovirulent strains typically induce more robust recruitment of these cellular populations compared to non-neurovirulent strains [188]. Additional systemic markers of infection include elevated serum amyloid A levels in EHV-1-infected horses [189], reflecting the broader inflammatory response associated with viral pathogenesis.

### 4.2. Viral Immune Evasion Strategies

To counteract immune cell recruitment and recognition, EHV-1 employs sophisticated immunomodulatory strategies through specific viral proteins. The viral chemokine-binding protein (vCKBP) gG binds and neutralizes chemokines like IL-8, effectively dampening neutrophil and monocyte migration [190,191]. Deletion studies of gG demonstrate enhanced inflammatory responses and increased immune cell migration to infected sites [86,186]. Additionally, glycoprotein gp2 exhibits similar chemokine-binding activity, while the early protein pUL56 performs dual immunoevasive functions by downregulating cell surface MHC I to evade cytotoxic T lymphocyte lysis and modulating chemokine expression to control leukocyte recruitment [192].

### 4.3. Leukocyte-Associated Viremia and Cell-to-Cell Spread

EHV-1 utilizes a sophisticated “Trojan horse” strategy, exploiting infected leukocytes to breach basement membranes and enter systemic circulation [178,179]. This immune evasion mechanism involves restricted expression of late viral glycoproteins (gC, gD) in infected leukocytes, protecting them from immune recognition despite the presence of neutralizing antibodies [193,194]. Viral replication remains highly restricted in primary carrier cells, particularly CD172a+ monocytic cells, with non-neurovirulent strains specifically delaying replication through epigenetic mechanisms involving silencing of viral gene expression by histone deacetylases (HDACs) [195,196]. Entry into these cells occurs inefficiently through sialic acid-containing receptors and integrin αvβ3 co-receptors, triggering entry via cholesterol-dependent, phagocytosis-like endocytic pathways [197].

T lymphocytes, particularly CD4+ cells, serve as important vehicles for viremia through distinct mechanisms. While complete viral replication cycles occur in these cells, glycoproteins aggregate at cell surfaces and virion assembly becomes impaired, preventing antibody recognition. Infectious virus transmission occurs efficiently through virological synapses upon contact with target cells, representing a form of cell-to-cell spread that effectively evades humoral immunity [188].

The adhesion of infected CD172a+ cells to endothelial cells is mediated by upregulated integrins (αVβ3, α4β1, αLβ2) and enhanced by pro-inflammatory cytokines in the local microenvironment [198]. Cell-to-cell contact subsequently activates viral replication in leukocytes and facilitates virion transfer to endothelial cells.

### 4.4. Endothelial Cell Infection and Interferon Suppression

Upon endothelial cell infection, EHV-1 actively suppresses host IFN responses to enable efficient replication. The virus disrupts IFN pathways at multiple levels by downregulating TLR3/4 and key interferon regulatory factors (IRF-3, IRF-7, IRF-9), inhibiting IRF-3 nuclear translocation, and degrading signaling components like tyrosine kinase 2 (TYK2) to suppress STAT1/STAT2 phosphorylation [199,200,201,202]. This comprehensive suppression of antiviral signaling, coupled with induction of pro-inflammatory states, promotes viral spread within endothelial tissues and contributes to the pathogenesis of clinical disease manifestations including abortion and myeloencephalopathy.

### 4.5. Pathophysiological Mechanisms of Major Disease Manifestations

#### 4.5.1. Abortion Pathophysiology

EHV-1-induced abortion represents a devastating reproductive complication characterized by complex vascular pathology at the uteroplacental interface. Following establishment of cell-associated viremia, virus-laden peripheral blood mononuclear cells (PBMCs), particularly CD172a+ monocytes and T lymphocytes, adhere to and transfer virus to endothelial cells lining the small arterioles of the pregnant uterus [198,203,204]. This adhesion is facilitated by upregulation of cellular adhesion molecules, including integrins αVβ3, α4β1, and αLβ2, on infected leukocytes and their corresponding receptors on endothelial cells—a process enhanced by pro-inflammatory cytokines in the local microenvironment [198,203].

EHV-1 infection of CD172a+ monocytic cells induces cellular changes that promote their adhesion to endothelial cells through a three- to five-fold increase in adhesion capacity [198]. Both cell-to-cell contact and secretion of soluble factors by endothelial cells, such as tumor necrosis factor-alpha (TNF-α), activate EHV-1 replication in CD172a+ cells, facilitating direct transfer of cytoplasmic viral material to adjacent endothelial cells [198] (Laval et al., 2015). This cell-associated transmission mechanism allows the virus to bypass antibody-mediated immune responses, explaining why vaccination alone cannot prevent viremia [203].

Upon endothelial cell infection and subsequent viral replication, widespread vasculitis develops, particularly affecting the small vascular networks supplying the glandular endometrium at the base of microcotyledons [205,206]. Endothelial cell necrosis triggers release of procoagulant factors, including tissue factor (thromboplastin), initiating intravascular thrombosis and ischemic necrosis of cotyledonary and intercotyledonary tissues [204,207]. This thrombo-ischemic cascade causes rapid progressive placental–endometrial separation immediately preceding fetal expulsion, with the fetus dying of anoxia as oxygen and nutrient supply is abruptly terminated [204,205,208].

Notably, widespread vascular endothelial damage may induce abortion even before detectable transplacental viral transmission to the fetus occurs [206,209]. The severity of abortion correlates with viral strain virulence, magnitude and duration of viremia, and hormonal influences on the uterine immune microenvironment during late pregnancy [210]. Additionally, endometrial resident lymphocytes may directly transfer virus to uterine endothelium, potentially explaining sporadic individual abortions or those occurring weeks to months after apparent resolution of viremia [182].

#### 4.5.2. Myeloencephalopathy Pathophysiology

Equine herpesvirus myeloencephalopathy (EHM) represents the most devastating neurological manifestation of EHV-1 infection, characterized by diffuse multifocal hemorrhagic myeloencephalopathy secondary to central nervous system (CNS) vasculitis and thrombosis [210,211]. The pathogenic sequence mirrors abortion pathophysiology but targets CNS vasculature specifically. Following establishment of cell-associated viremia, infected leukocytes transport virus to CNS endothelial cells lining small arteries and arterioles throughout the brain, brainstem, and spinal cord [203].

The neuropathogenic D752 variant of EHV-1, characterized by a single nucleotide polymorphism in the DNA polymerase gene (ORF30), demonstrates 10- to 100-fold higher viremia levels, longer persistence, and preferential CD4+ T lymphocyte tropism compared to non-neuropathogenic N752 strains, facilitating more efficient CNS invasion [210,212,213]. This SNP at position 2254 of ORF30 results in an amino acid variation (N752/D752) that significantly influences neuropathogenic potential [214,215]. However, recent surveillance data demonstrates that the N752 genotype has become the predominant variant detected not only in respiratory disease (87.5%) and abortion (80%) cases, but also in EHM cases (74.3%), challenging earlier assumptions about genotype-disease associations [216]. Upon close contact or adhesion between virus-infected PBMCs and CNS endothelial cells, cell-to-cell viral transmission occurs, bypassing antibody-mediated immune responses [203]. Infected endothelial cells undergo lytic infection, triggering vasculitis of CNS arterioles. This vasculitis is followed by intravascular thrombosis and focal infarction, ultimately resulting in ischemic degeneration manifesting as areas of malacia (tissue softening) in both white and gray matter [210,211].

Histopathologic examination reveals discrete lesions comprising vasculitis with endothelial cell damage and perivascular cuffing, thrombus formation, hemorrhage, and in advanced cases, extensive malacial regions distributed throughout the brain and spinal cord [217]. Critically, there is no evidence of direct neuronal invasion by the virus; instead, neurological deficits result entirely from ischemic damage secondary to vascular thrombosis [210,218].

Cerebrospinal fluid analysis typically reveals xanthochromia, elevated protein concentrations (100–500 mg/dL), and increased albumin quotient, reflecting vasculitis and protein leakage, though white blood cell counts usually remain normal [217]. The acute onset and rapid progression of clinical signs (within 24–48 h) reflect the fulminant nature of CNS vascular damage, with ataxia, paresis, urinary incontinence, and in severe cases, recumbency representing the multifocal distribution of ischemic spinal cord lesions [133,218]. Studies investigating EHV-1-induced encephalomyelitis have demonstrated that intranasal infection with highly neurovirulent strains results in fulminant neurological disease characterized by high viral brain titers, severe encephalitis, and extensive monocyte and CD8+ T cell infiltration. Brain tissue analysis reveals upregulation of chemokines CCL2, CCL3, CCL4, CCL5, CXCL2, CXCL9, and CXCL10, with more virulent strains showing higher levels correlating with increased inflammatory cell numbers [182,219].

#### 4.5.3. Equine Multinodular Pulmonary Fibrosis Pathophysiology

Equine multinodular pulmonary fibrosis (EMPF) represents a progressive fibrosing interstitial lung disease strongly associated with gammaherpesvirus EHV-5 infection, though the exact pathophysiological mechanisms remain incompletely understood [220,221]. Unlike EHV-1-mediated diseases that follow acute viremic dissemination, EMPF appears to develop during latent or persistent EHV-5 infection through mechanisms involving chronic viral stimulation of fibrotic pathways [222,223]. Affected horses demonstrate loss of functional pulmonary parenchyma due to extensive nodular interstitial fibrosis characterized by multiple well-demarcated nodular regions of fibrosis with mixed inflammatory cell infiltration [132,221].

Experimental inoculation studies have confirmed that EHV-5 isolated from spontaneous EMPF cases can induce pulmonary fibrosis when inoculated into clinically normal horses, providing strong evidence for viral causation [223]. This landmark study represents the first demonstration that a gammaherpesvirus can induce lung fibrosis in its natural host species without additional known lung injury. The pathogenic process involves viral infection of alveolar epithelial cells and pulmonary lymphocytes, with subsequent cell-to-cell viral spread within alveolar tissues [222,223].

Critically, the disease involves massive induction and accumulation of myofibroblasts—α-smooth muscle actin-positive cells that produce excessive extracellular matrix proteins, particularly collagen [223]. These myofibroblasts appear within alveolar walls in mildly affected regions, with numbers and density increasing proportionally with fibrosis severity, ultimately becoming embedded within interstitial collagen deposits and beneath epithelial cells lining airspaces [223].

The mechanism by which EHV-5 triggers fibrosis likely involves complex virus–host interactions during latency, with viral replication combined with host-specific predisposing factors eventually triggering fibrotic cascades [222,224]. Recent experimental ex vivo and in vitro studies have shown that infected equine T and B lymphocytes could act as lifelong latency reservoirs for the virus and play a role in the development of disease [224]. Interestingly, not all EHV-5 strains appear pathogenic, with nucleic acid sequence analysis revealing that not all amplicons from inoculated horses matched the original inoculation strains, suggesting that specific viral genomic variations confer fibrogenic properties [223]. The disease parallels human idiopathic pulmonary fibrosis associated with Epstein–Barr virus (another gammaherpesvirus), suggesting shared pathogenic mechanisms across species [224].

Clinical presentation includes pyrexia, progressive weight loss, tachypnea, dyspnea, hypoxemia, neutrophilia, and hyperfibrinogenemia, with thoracic imaging revealing characteristic diffuse nodular interstitial patterns [132,221,225]. The prognosis remains poor, with most affected horses succumbing to progressive respiratory failure despite treatment attempts [226].

## 5. Treatment of EHV Infection

Currently, no effective treatment has been established for EHV infection [22]. Despite numerous attempts to repurpose antiviral agents commonly used for other herpesvirus infections, most conventional antivirals have demonstrated limited or no clinical efficacy against EHV, particularly in cases of neurological disease.

### 5.1. Conventional Antivirals with Limited Efficacy

Acyclovir and its prodrug valacyclovir, which are highly effective against human alphaherpesviruses, have shown disappointing results in treating EHV-1 and EHV-4 infections. While these nucleoside analogs inhibit viral DNA polymerase in vitro, clinical studies have failed to demonstrate significant therapeutic benefit in horses with EHV-1 myeloencephalopathy or respiratory disease. The limited efficacy may be attributed to several factors, including poor oral bioavailability in horses, inadequate tissue penetration to sites of viral replication (particularly the central nervous system), and potential differences in viral thymidine kinase activity compared to human herpesviruses. Notably, acyclovir treatment has shown some effectiveness in terminating EHV-3 excretion within 8 days after onset in stallions with equine coital exanthema (ECE), a venereally-transmitted mucocutaneous disease characterized by papules, vesicles, pustules and ulcers on the external genital organs [55,227,228,229]. However, this specific application to EHV-3 does not translate to broader efficacy against the more clinically significant EHV-1 and EHV-4 infections.

### 5.2. Immunomodulators and Their Uncertain Impact

Various immunomodulatory approaches have been explored for EHV infection management, including the use of inactivated Parapoxvirus ovis (iPPVO)-based immunomodulators in the United States [22,230]. The theoretical rationale for immunomodulation includes enhancement of innate immune responses through stimulation of interferon production and potential reduction of viral shedding [230,231]. One randomized controlled study demonstrated that iPPVO treatment reduced clinical signs and EHV-1 viral shedding in an experimental challenge model [22]. However, a comprehensive systematic review of pharmacologic interventions for EHV-1 concluded that most studies reported either no benefit or minimal efficacy of immunomodulatory treatments, with minimal or limited benefit as either prophylactic or post-exposure treatment in mitigating EHV-1-associated disease outcomes [230]. The 2024 ACVIM consensus statement affirmed that there is no evidence that pharmacologic treatments given after the onset of clinical signs of EHV-1 infection prevent or affect the development or course of EHM [232]. Furthermore, iPPVO (Zylexis, Zoetis, USA) has been discontinued and is no longer available in the United States [233]. The risk–benefit profile of immunostimulation during active herpesvirus infection remains controversial, as excessive immune activation could potentially exacerbate endothelial damage and thrombotic complications characteristic of severe EHV-1 disease.

### 5.3. Emerging Therapeutic Candidates

Despite the limitations of conventional approaches, recent research has identified several compounds with promising antiviral activity against herpes virus infections in in vitro studies and murine models [234]. These emerging therapeutic candidates demonstrate diverse mechanisms of action and represent potential avenues for future clinical development.

Among the most promising compounds are bioactive alkaloids, particularly berbamine (BBM), which has demonstrated significant therapeutic potential against EHV-1. In vitro studies revealed that BBM effectively suppresses multiple stages of the viral life cycle, including viral entry into host cells, viral DNA replication, and virion release. Subsequent in vivo studies in murine models confirmed that BBM treatment significantly reduced EHV-1-induced pathological damage in brain and lung tissues while decreasing animal mortality rates [234]. Similarly, cepharanthine, a natural bisbenzylisoquinoline alkaloid extracted from Stephania cepharantha Hayata, has shown dose-dependent inhibitory effects against EHV-8 infection in NBL-6 and RK-13 cell lines. Mechanistic analysis revealed that cepharanthine reduces EHV-8-induced oxidative stress through activation of AMPK and Nrf2/HO-1 signaling pathways, with in vivo studies demonstrating significant amelioration of lung pathology and reduced oxidative stress in EHV-8-infected mice [235]. Consistently, another study found that celastrol prevents EHV-8 infection in vitro by activating the Nrf2/HO-1 signaling pathway and relieving oxidative stress [236].

Complementing these alkaloid-based approaches, blebbistatin represents a novel therapeutic strategy targeting cellular mechanisms essential for viral infection. Myosin II ATPase activity is crucial for EHV infection as it drives the cytoskeletal rearrangements, membrane dynamics, and intracellular transport processes required for successful viral cellular entry, trafficking to replication sites, and egress from infected cells. Studies demonstrated that blebbistatin exhibited dose-dependent inhibition of EHV-8 infection in RK-13 and MDBK cell lines by disrupting viral entry through myosin II ATPase modulation, with in vivo studies confirming significant reduction in viral replication and amelioration of pulmonary pathology in infected mice [139].

The therapeutic landscape is further enriched by compounds targeting host antioxidant and immunomodulatory pathways. Hyperoside, isolated from Rhododendron brachycarpum G. Don, exhibits potent antiviral activity against EHV-8 in multiple cell lines, including RK-13 (rabbit kidney cells), MDBK (Madin-Darby bovine kidney), and NBL-6 (E. Derm cells). Mechanistically, hyperoside activates the JNK/Nrf2/Keap1 pathway, inducing heme oxygenase-1 expression, reducing oxidative stress, and triggering antiviral interferon responses, with in vivo studies confirming significant reduction in pulmonary pathology in EHV-8-infected mice [237]. This heme oxygenase-1 pathway is also targeted by cobalt protoporphyrin, which has demonstrated significant inhibition of EHV-8 replication in susceptible cell lines and murine models through HO-1-mediated type I interferon responses [238].

These findings collectively suggest that targeting multiple viral replication stages, cellular entry mechanisms, and host antioxidant pathways may provide effective therapeutic strategies against EHV infections. The diverse mechanisms of action demonstrated by these emerging compounds offer potential for combination therapies that could enhance antiviral efficacy while minimizing the risk of viral resistance development. However, it is important to note that most of these promising candidates have only been evaluated in cell culture systems and murine models, primarily against EHV-8 rather than the clinically more significant EHV-1 and EHV-4. Translation of these findings to equine clinical applications will require rigorous evaluation of safety, pharmacokinetics, and clinical efficacy in the target species. Until such evidence becomes available, EHV management continues to rely primarily on biosecurity measures, vaccination programs, and supportive care rather than specific antiviral therapy.

## 6. Efficacy and Limitations of Current EHV Vaccination Strategies

### 6.1. Fundamental Properties of Available EHV Vaccines

EHV vaccines encompass diverse platforms with distinct immunological properties, mechanisms of action, and efficacy profiles [239]. Understanding these fundamental differences is essential for informed vaccination strategies and development of improved vaccines.

#### 6.1.1. Inactivated (Killed) Vaccines

Inactivated EHV vaccines represent the most widely used vaccine platform globally and contain chemically or physically inactivated whole virus particles combined with adjuvants to enhance immunogenicity [240,241,242]. These vaccines induce primarily humoral (antibody-mediated) immune responses, generating virus-neutralizing antibodies and EHV-specific IgG responses, particularly IgG1 and IgG4/7 isotypes [243]. The high-antigen-load inactivated EHV-1 vaccines licensed for abortion prevention demonstrate superior performance compared to respiratory-only formulations, producing higher antibody titers and some evidence of cellular immune responses [244,245,246].

Inactivated vaccines offer excellent safety profiles with no risk of reversion to virulence or shedding of vaccine virus, making them particularly suitable for pregnant mares and immunocompromised animals [211]. They provide consistent, reproducible immune responses and demonstrate relative stability during storage. For abortion prevention specifically, inactivated vaccines administered during months 3, 5, 7, and 9 of gestation have demonstrated measurable efficacy in reducing abortion storms in pregnant mare populations [247]. Research has demonstrated that intramuscular administration of inactivated EHV-1/4 vaccines successfully induces robust mucosal antibody responses at upper respiratory tract entry sites, confirming that systemic vaccination generates localized immunity at sites where viral infection initially establishes [248]. The vaccination protocol primarily stimulates protective IgG4/7 antibodies both systemically and mucosally, with serum antibody levels remaining elevated throughout extended study periods, and mucosal antibodies demonstrating consistent increases following repeated vaccine doses [248].

However, these vaccines stimulate only humoral immunity without robust cell-mediated (Th1-type) responses, potentially providing insufficient protection against respiratory infection and viral replication [245]. The immunity generated is relatively short-lived, lasting approximately 3–6 months, which necessitates frequent revaccination schedules [211,247]. Despite their widespread use, inactivated vaccines do not prevent infection or significantly reduce viral shedding, allowing vaccinated horses to harbor and transmit virus to susceptible animals, thereby perpetuating transmission chains within populations [211,242]. Individual animals maintain susceptibility to infection despite adherence to established vaccination protocols [247].

Clinical observations have revealed complex relationships between vaccination status and disease progression patterns. Paradoxically, appropriately vaccinated horses demonstrated extended hospitalization periods (15.5 days) compared to inadequately vaccinated animals (12.5 days), suggesting that vaccination modulates disease trajectory and recovery patterns rather than providing complete protection against severe disease onset [104]. Despite these extended hospitalization requirements, appropriately vaccinated horses demonstrated superior immune responses, maintaining elevated lymphocyte counts within 24 h of admission and throughout the entire hospitalization period. This preservation of critical immune function during acute disease phases indicates that vaccination favorably modulates immune responses, potentially resulting in more robust yet prolonged immune-mediated recovery processes that necessitate extended veterinary monitoring [104].

#### 6.1.2. Modified-Live Virus (MLV) Vaccines

Modified-live vaccines utilize attenuated EHV-1 strains engineered through targeted gene deletions to reduce virulence while maintaining immunogenicity [246]. The commercially available MLV vaccines, including Rhinomune in the United States and Prevaccinol in Europe, are based on the RacH strain with deletions in open reading frames ORF1, ORF2, and ORF67 [211,242,244]. These vaccines more closely mimic natural infection compared to inactivated vaccines, thereby stimulating both humoral and cell-mediated immunity, including cytotoxic T lymphocyte (CTL) responses that are critical for clearing infected cells [244,245,246].

MLV vaccines induce significantly lower nasal virus shedding following challenge infection compared to inactivated vaccines, potentially reducing viral transmission at population levels [245]. They generate balanced immune responses characterized by both antibody production and Th1-biased cellular immunity, evidenced by lower IgG(T)/IgGa and IgG(T)/IgGb ratios that suggest a cytotoxic immune response bias [245,246]. Notably, experimental deletion mutants such as Ab4ΔORF2 have demonstrated the ability to completely prevent viral replication in the upper respiratory tract, eliminate nasal shedding, and prevent cell-associated viremia in fully protected horses, representing a significant advancement over current commercial products [249].

Despite these immunological advantages, MLV vaccines carry theoretical risks of reversion to virulence, vaccine virus shedding, or establishment of latency, although such events have not been documented with current commercial products [211]. These vaccines typically generate lower virus-neutralizing antibody titers compared to inactivated vaccines, which may concern practitioners accustomed to relying on serological markers as indicators of protection [245,246]. Some individual animals may exhibit stronger vaccine reactions, including localized swelling at injection sites or transient fever. Importantly, current MLV vaccines are licensed only for respiratory disease prevention and are not approved for use in pregnant mares due to theoretical abortion risks, thus limiting their application in breeding populations where abortion prevention is a primary concern [211].

Recent research has revealed an additional concern regarding vaccination frequency. Frequent EHV vaccination with inactivated commercial vaccines at short intervals of 60–90 days can result in declining antibody values and cellular immunity despite additional vaccine boosts, a phenomenon termed “adverse immunity to EHV vaccination” [245]. This finding has important implications for vaccination protocols, particularly in high-risk situations where frequent boosting might seem intuitively beneficial but may actually compromise protective immunity.

#### 6.1.3. Recombinant Vector Vaccines

Recombinant vaccines utilize heterologous viral vectors, particularly canarypox virus or modified vaccinia Ankara, that are engineered to express specific EHV glycoproteins while retaining the ability to replicate in avian cells but not in mammalian cells [211]. This platform combines the safety advantages of inactivated vaccines with the enhanced cellular immunity induction characteristic of MLV vaccines. These vaccines offer excellent safety profiles because the vector cannot replicate in horses, thereby eliminating concerns about reversion to virulence or establishment of latency. They successfully induce both humoral and cell-mediated immune responses, including strong interferon-gamma production and CTL activation. Additionally, the vaccines can be engineered to express multiple antigens simultaneously or combined with other vaccines, such as concurrent administration of recombinant canarypox equine influenza vaccine with inactivated EHV vaccine. However, recombinant vaccines remain largely experimental platforms with limited commercial availability for EHV. A significant concern is that vector-specific immunity may develop upon repeated administration, potentially reducing the efficacy of boost responses over time. Furthermore, the manufacturing complexity and associated costs substantially exceed those of traditional vaccine platforms, limiting their widespread adoption.

#### 6.1.4. DNA and Subunit Vaccines

DNA vaccines consist of plasmids encoding specific EHV antigens, typically glycoproteins gB, gC, or gD, that are delivered intramuscularly to induce immune responses through in vivo antigen expression. Subunit vaccines, in contrast, contain purified viral proteins that directly stimulate the immune system. Both platforms offer precise antigen selection and excellent safety profiles. However, these vaccine types have demonstrated variable and often poor immunogenicity in horses, requiring sophisticated adjuvant systems or novel delivery mechanisms to enhance immune responses [211]. Consequently, neither DNA nor subunit vaccines have achieved widespread commercial success for EHV prevention, remaining primarily in the experimental and research domains.

### 6.2. Vaccine Efficacy and Population-Level Limitations

Despite providing measurable individual immunological benefits, current EHV vaccines demonstrate fundamental limitations in preventing infection and controlling disease transmission at population levels. A comprehensive meta-analysis examining vaccination efficacy against EHV-1 revealed that vaccination generally results in only slight improvements in clinical and virological outcomes, although these improvements did not reach statistical significance in many parameters [28]. The analysis of 36 randomized controlled trials showed that vaccinated horses experienced a reduced relative risk of pyrexia (0.468, 95% confidence interval: 0.318–0.688), but this finding was accompanied by severe between-trial heterogeneity and significant publication bias, limiting the strength of conclusions.

These findings reflect the inherent challenge that EHV vaccines, regardless of platform, induce incomplete immunity that may protect against severe disease manifestations but fails to prevent infection or significantly reduce viral shedding [211,216]. Vaccinated horses continue to develop cell-associated viremia and can transmit virus to susceptible animals, thereby perpetuating outbreak chains within populations [133,203]. The short duration of vaccine-induced immunity, lasting approximately 3–6 months, combined with the ubiquity of latently infected horses capable of viral reactivation, creates persistent reinfection pressure that overwhelms vaccine-induced protection [210,211].

Most critically, an analysis of twelve EHV-1 outbreak studies revealed no statistically significant difference between the basic reproduction number (R_0_) in non-vaccinated herds and the reproduction number (Rv) in fully vaccinated herds (*p* = 0.15) [216]. More significantly, Rv in herds where all horses were vaccinated remained substantially above 1, indicating that vaccination alone was insufficient to prevent sustained viral transmission during natural outbreaks [250]. These findings demonstrate that while vaccination provides individual benefits regarding disease severity and immune responses, it exhibits limited effectiveness as a standalone population-level intervention for EHV-1 transmission control, a conclusion supported by multiple independent studies [251,252,253,254].

In outbreak situations characterized by high viral loads and the presence of “superspreader” horses that shed large quantities of virus, even the immunity of vaccinated horses can become overwhelmed [255]. Recent outbreak investigations have identified horses that shed substantially more virus than others despite showing few or no clinical signs, presenting a significant challenge to outbreak control regardless of vaccination status [103]. While vaccines can reduce the severity of clinical disease and may decrease nasal viral shedding to some degree, they do not prevent the establishment of viremia or eliminate the potential for horses to transmit infection to other animals [28,216].

The emergence of the novel H752 genotype further complicates vaccination strategies and outbreak management. This variant, first identified in Europe and subsequently detected in North American outbreaks, does not fit into the previously recognized D752 or N752 genotype classifications and can result in negative EHV-1 test results when diagnostic laboratories rely exclusively on genotype-specific assays [255]. This diagnostic challenge emphasizes the need for multi-gene molecular approaches rather than relying solely on single nucleotide polymorphism detection at the ORF30 position.

Consequently, effective EHV control requires integrated, multi-faceted approaches that combine vaccination for individual disease mitigation with rigorous biosecurity measures. These measures must include movement restrictions, strict isolation protocols for incoming horses (minimum 21 days), environmental disinfection, twice-daily temperature monitoring to enable early outbreak detection, and minimizing stress-induced viral reactivation through appropriate management practices [43,116,211]. Vaccination alone cannot ensure population-level protection, and unrealistic expectations of vaccine performance may inadvertently compromise outbreak control efforts by creating false confidence in protection [211,216]. The equine industry and governing bodies have increasingly recognized these limitations, implementing more stringent biosecurity protocols at competition venues and requiring documentation of vaccination status as part of comprehensive disease management strategies [43,216].

Future vaccine development should target conserved epitopes in glycoproteins B, D, and H/L complex that are functionally constrained across EHV-1/-4/-8. Efficacy evaluation must occur in high-transmission settings such as racing stables and donkey milk farms to determine if next-generation vaccines can achieve the population-level transmission control that current vaccines fail to provide.

## 7. Conclusions and Future Research Directions

Equine herpesvirus infections constitute a multifaceted global health challenge that significantly impacts equine welfare and industry economics worldwide. The taxonomic diversity of nine EHV species, coupled with their sophisticated molecular pathogenesis mechanisms and complex glycoprotein networks, creates substantial obstacles for effective disease control. While global surveillance has documented widespread distribution and diverse clinical manifestations across all continents, current therapeutic and preventive approaches remain inadequate. The limited efficacy of existing vaccines and absence of effective treatments highlight critical gaps in our disease management capabilities. The virus’s sophisticated immune evasion strategies, particularly the exploitation of infected leukocytes for systemic dissemination, demonstrate the evolutionary adaptation of these pathogens to their equine hosts. Comprehensive integration of advanced molecular diagnostics, innovative therapeutic development, and enhanced biosecurity measures is essential for developing effective control programs that protect both individual animal health and broader industry interests.

Future research should prioritize development of next-generation vaccines targeting conserved viral epitopes across multiple EHV species, potentially utilizing mRNA or viral vector platforms to enhance mucosal immunity. Advanced therapeutic discovery programs should focus on a combination of antiviral strategies targeting multiple viral replication stages and host cellular pathways simultaneously. Comprehensive genomic surveillance networks are needed to monitor viral evolution, emergence of new variants, and cross-species transmission events. Investigation of host genetic factors influencing susceptibility and disease severity could identify biomarkers for personalized prevention strategies. Integration of One Health approaches examining human–animal–environmental interfaces will be crucial for understanding transmission dynamics. Additionally, development of rapid point-of-care diagnostic tools and artificial intelligence-based prediction models for outbreak management represents critical technological advances needed for effective disease control in modern equine populations.

## Figures and Tables

**Table 1:** Classification of EHVs.

Virus Type	Subfamily/Genus	Primary Host	References
EHV-1	Alphaherpesvirinae/Varicellovirus	Donkeys and Horses	[49,50,51,52]
EHV-3	Alphaherpesvirinae/Varicellovirus	Horses, donkeys (rare)	[30,53,54,55,56]
EHV-4	Alphaherpesvirinae/Varicellovirus	Horses, Donkeys	[57,58,59,60]
EHV-8	Alphaherpesvirinae/Varicellovirus	Horses (Rare), Donkeys	[61,62]
EHV-9	Alphaherpesvirinae/Varicellovirus	Horses (Rare)	[63]
EHV-2	Gammaherpesvirinae/Percavirus	Horses, Donkeys (Rare)	[64,65]
EHV-5	Gammaherpesvirinae/Percavirus	Horses, Donkeys	[65,66]
EHV-7	Gammaherpesvirinae/Varicellovirus	Donkeys (Rare) and Horses (rare)	[67]

## Data Availability

No new data were created or analysed in this study.
